# Differentially Expressed mRNAs and Their Long Noncoding RNA Regulatory Network with *Helicobacter pylori*-Associated Diseases including Atrophic Gastritis and Gastric Cancer

**DOI:** 10.1155/2020/3012193

**Published:** 2020-11-17

**Authors:** Songyi Liu, Honghao Yin, Shuwen Zheng, Aining Chu, Yizhi Li, Chengzhong Xing, Yuan Yuan, Yuehua Gong

**Affiliations:** ^1^Tumor Etiology and Screening Department of Cancer Institute and General Surgery, The First Hospital of China Medical University, Shenyang 110001, China; ^2^Key Laboratory of Cancer Etiology and Prevention in Liaoning Education Department, The First Hospital of China Medical University, Shenyang 110001, China; ^3^Key Laboratory of GI Cancer Etiology and Prevention in Liaoning Province, The First Hospital of China Medical University, Shenyang 110001, China

## Abstract

**Background:**

*Helicobacter pylori* (*Hp*) infection is the strongest risk factor for gastric cancer (GC). However, the mechanisms of *Hp*-associated GC remain to be explored.

**Methods:**

The gene expression profiling (GSE111762) data were downloaded from the GEO database. Differentially expressed genes (DEGs) between normal samples (NO) and *Hp*-atrophic gastritis (GA) or *Hp*-GA and *Hp*-GC were identified by GEO2R. Gene Ontology and pathway enrichment analysis were performed using the DAVID database. lncRNA-TF-mRNA and ceRNA regulation networks were constructed using Cytoscape. The cross-networks were obtained by overlapping molecules of the above two networks. GSE27411 and GSE116312 datasets were employed for validation.

**Results:**

DEGs between NO and *Hp*-GA are linked to the activity of inward rectifying potassium channels, digestion, etc. DEGs between *Hp*-GA and *Hp*-GC were associated with digestion, positive regulation of cell proliferation, etc. According to the lncRNA-TF-mRNA network, 63 lncRNAs, 12 TFs, and 209 mRNAs were involved in *Hp*-GA while 16 lncRNAs, 11 TFs, and 92 mRNAs were contained in the *Hp*-GC network. In terms of the ceRNA network, 120 mRNAs, 18 miRNAs, and 27 lncRNAs were shown in *Hp*-GA while 72 mRNAs, 8 miRNAs, and 1 lncRNA were included in the *Hp*-GC network. In the cross-network, we found that immune regulation and differentiation regulation were important in the process of NO-GA. Neuroendocrine regulation was mainly related to the process of GA-GC. In the end, we verified that CDX2 plays an important role in the pathological process of NO to *Hp*-GA. Comparing *Hp*-GA with *Hp*-GC, DEGs (FPR1, TFF2, GAST, SST, FUT9, and SHH), TF, and GATA5 were of great significance.

**Conclusions:**

We identified the DEGs, and their lncRNA regulatory network of *Hp*-associated diseases might provide insights into the mechanism between *Hp* infection and GC. Furthermore, in-depth studies of the molecules might be useful to explore the multistep process of gastric diseases.

## 1. Introduction

The current study assumed that the *Helicobacter pylori* (*Hp*) infection was closely associated with gastric cancer (GC). Nonatrophic gastritis, atrophic gastritis (GA), intestinal metaplasia (IM), and dysplasia were included in the pathological process which led to the GC ultimately [1, 2]. In the process above, the risk of *Hp*-positive GA patients to develop GC is 6.4-11.8 times as high as the noninfected ones [3]. Therefore, searching for the GA and GC molecular markers associated with *Hp* infection is of great significance to the early diagnosis and reversal of GC.

After setting on the epithelium and glands of the gastric mucosa, *Hp* arouses inflammation through a variety of adhesives and virulence factors, leading to the changes of signaling pathways in the host [4]. Also, *Hp* infection increases oxidative stress by inducing apoptosis and then disrupts cellular integrity and produces inflammation-related tumors [5]. However, the previous studies on the mechanism of *Hp* infection-related gastric diseases were limited to a single molecule or a signal pathway. Gene expression and the multistage pathological process of *Hp*-related gastric diseases were not displayed enough formerly. Thus, to reveal the development of gastric diseases, a systematic understanding of *Hp*-related gastric precancerous diseases and gene expression alternations in GC is in urgent need.

Regulation of gene expression includes transcription level and posttranscriptional levels. Transcription factor (TF) is the main regulator in the transcriptional level, which can bind to the DNA region of the enhancers, or promoters adjacent to the target gene [6, 7]. Noncoding RNAs (ncRNAs) are proven to be important epigenetic regulators in the posttranscriptional level [8, 9]. MicroRNA (miRNA) belongs to small ncRNAs, inducing gene degradation or inhibiting translation by binding to mRNA. lncRNAs are endogenous cellular RNA transcripts longer than 200 nucleotides in length [10], becoming cancer essential regulators with tissue-specific patterns and cell-specific patterns [10–13]. Abnormally expressed miRNAs and lncRNAs have been regarded as promising diagnostic and prognostic biomarkers, existing not only in GC but also in other tumors [14, 15]. lncRNAs can inhibit miRNA in the cytoplasm as a competitive endogenous RNA (ceRNA). lncRNAs can regulate the activity of TF in the nucleus as well [16]. Studies have reported that the differentially expressed lncRNAs, identified in *Hp*-infected tissue of GC, could be involved in the development of *Hp*-related gastric diseases [17]. However, the research on *Hp*-related transcription and noncoding regulation is still in its infancy.

As the availability of multilevel expression data, the integration of large datasets such as Gene Expression Omnibus (GEO) offers new opportunities for the public to comprehensively understand the cancer development [18–20]. The research, therefore, is intended to construct mRNA-lncRNA regulatory networks among normal, GA, and GC with *Hp* infection. Our study may provide insights into the mechanism between *Hp* infection and GC.

## 2. Materials and Methods

### 2.1. Data Acquisition of Differentially Expressed Genes (DEGs)

GSE111762, an lncRNA/mRNA analysis set, downloaded from the GEO database, included three normal human gastric mucosal tissues (NO), six *Hp*-positive GA patients (*Hp*-GA), and six *Hp*-positive GC patients (*Hp*-GC). GEO2R (http://www.ncbi.nlm.nih.gov/geo/geo2r/) [21] was used to screen DEGs between NO and *Hp*-GA or *Hp*-GA and *Hp*-GC, respectively. The falsediscoveryrates(FDR) < 0.05 and ∣logFC | >2 were considered statistically significant.

Human lncRNA and protein-coding gene annotations were directly downloaded from GENECODE v22. All of the categories in the “long non-coding RNA gene annotation” GTF file were considered to be lncRNAs. Duplicate probes were removed.

### 2.2. GO and KEGG Pathway Enrichment Analysis

Gene Ontology (GO) analysis is the primary bioinformatics tool to unify the characterization of genes and gene products [22]. GO contains three categories of terms, including cellular component, molecular function, and biological process. KEGG is a set of databases containing information about genomes, biological pathways, diseases, and chemicals [23]. DAVID (https://david.ncifcrf.gov/) is a bioinformatics data resource with an integrative bioinformation database and analysis tools and benefits to discover the biological meaning behind genes [24]. DEGs were enriched and analyzed by DAVID for GO and KEGG pathways, respectively. *P* < 0.05 was considered statistically significant.

### 2.3. Construction of the lncRNA-TF-mRNA Regulatory Network

The correlation coefficient and significance thresholds were set at 0.95 and 0.001 in the comparison between NO and *Hp*-GA while 0.85 and 0.001 were set between *Hp*-GA and *Hp*-GC. The protein-protein interaction (PPI) network was constructed using the STRING online database [25]. TFs were annotated using the TF checkpoint [26]. The regulatory relationships among mRNAs, TFs, and lncRNAs were visualized using the Cytoscape software (version 3.4.0). The CentiScaPe app was used to analyze the computing network's topological property [27]. lncRNAs, TFs, and mRNAs were ranked to obtain the key genes based on the degree size.

### 2.4. Construction of the ceRNA Regulatory Network

miRWalk is a database to predict miRNA target genes [28]. It integrates miRDB, miRTarbase, and TargetScans. Considering the inverse correlations, miRWalk was applied to observe the interaction between miRNAs and mRNAs. The overlapping miRNAs were further analyzed. DIANA is a database that predicts the association between miRNAs and lncRNAs. miRNA-targeted lncRNAs were similarly predicted via DIANA. Furthermore, the predicted lncRNAs were intersected with the lncRNAs with different expression in the GSE111762 dataset. Besides, the Cytoscape software took advantage of building the ceRNA network. The number of each interaction was calculated to identify key genes in the network as well.

### 2.5. Construction of the Cross-Network

The overlapping genes of the above lncRNA-TF-mRNA network and ceRNA network were synthesized. Meanwhile, a crossover network was constructed using the Cytoscape software.

### 2.6. GEO Dataset Analysis for the Validation

**Figure 1:**
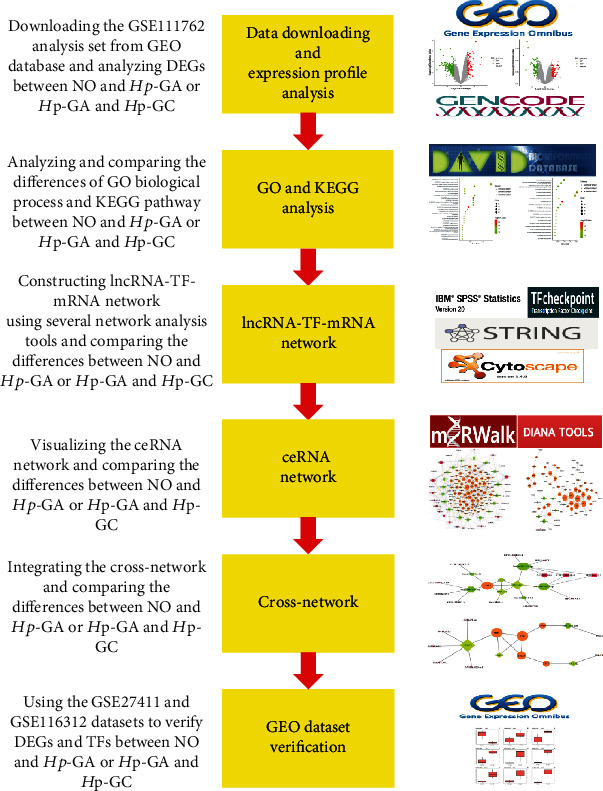
General workflow of the DEG screening and network construction.

The GSE27411 dataset included six *Hp*-negative normal gastric tissue samples and six *Hp*-positive GA samples. Three *Hp*-positive GA samples and three *Hp*-positive GC samples were included in the GSE116312 dataset. In this study, the GSE27411 dataset was used to analyze the selected DEGs and TFs between NO and *Hp*-GA. Besides, we selected the GSE116312 dataset to analyze the key DEGs and TFs screened by the results above. The entire workflow is shown in Figure 1.

### 2.7. Statistical Analyses

Coexpression relationships between the lncRNAs and mRNAs were estimated by Spearman's correlation test. FDR was also calculated to correct the *P* value for multiple testing. Unless otherwise stated, statistical significance was considered *P* < 0.05.

## 3. Results

### 3.1. Screening of *Hp*-GA- and *Hp*-GC-Related DEGs

#### 3.1.1. Screening of *Hp*-GA-Related DEGs

**Figure 2:**
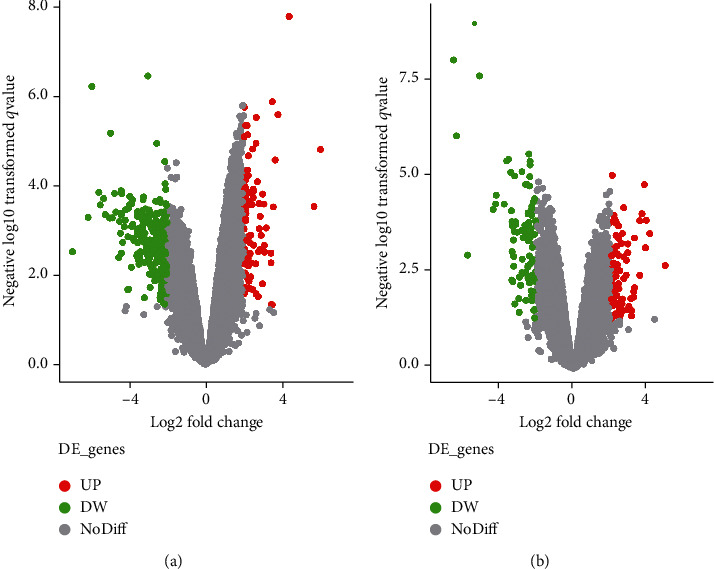
Volcano plot of the DEGs in the gene expression dataset GSE111762. (a) Red color is indicative of upregulated genes and green color of downregulated genes in normal vs. *Hp*-GA. (b) Red color is indicative of upregulated genes and green color of downregulated genes in *Hp*-GA vs. *Hp*-GC. Gray color indicates genes that are not differentially expressed in a statistically significant manner (the cutoff values of FDR < 0.05 and ∣logFC | >2).

A total of 389 DEGs were obtained between NO and *Hp*-GA, which showed 88 upexpressed and 301 downexpressed genes in GA (Figure 2(a)). By removing 18 duplicate probes, 81 upexpressed and 290 downexpressed genes were finally left (Table S1). Among them, there were 7 highly expressed lncRNAs, 67 downexpressed lncRNAs, 74 high-level mRNAs, and 223 low-level mRNAs. The top 5 of DEG names are shown in Tables 1(a) and 2(a), respectively.

#### 3.1.2. Screening of *Hp*-GC-Related DEGs

In the comparison between *Hp*-GA and *Hp*-GC, a total of 187 DEGs showed 88 upexpressed genes and 99 downexpressed genes in GC (Figure 2(b)). By removing 14 duplicate probes, 84 highly expressed genes and 89 low-expression genes were finally left (Table S2). Among them, there were 11 high-level lncRNAs, 9 low-level lncRNAs, 73 high-level mRNAs, and 80 low-level mRNAs. The top 5 of DEG names are shown in Tables 1(b) and 2(b), respectively.

### 3.2. Enrichment Analysis of GO and KEGG Pathways of DEGs

#### 3.2.1. Functional Enrichment Analysis of *Hp*-GA-Related DEGs

**Figure 3:**
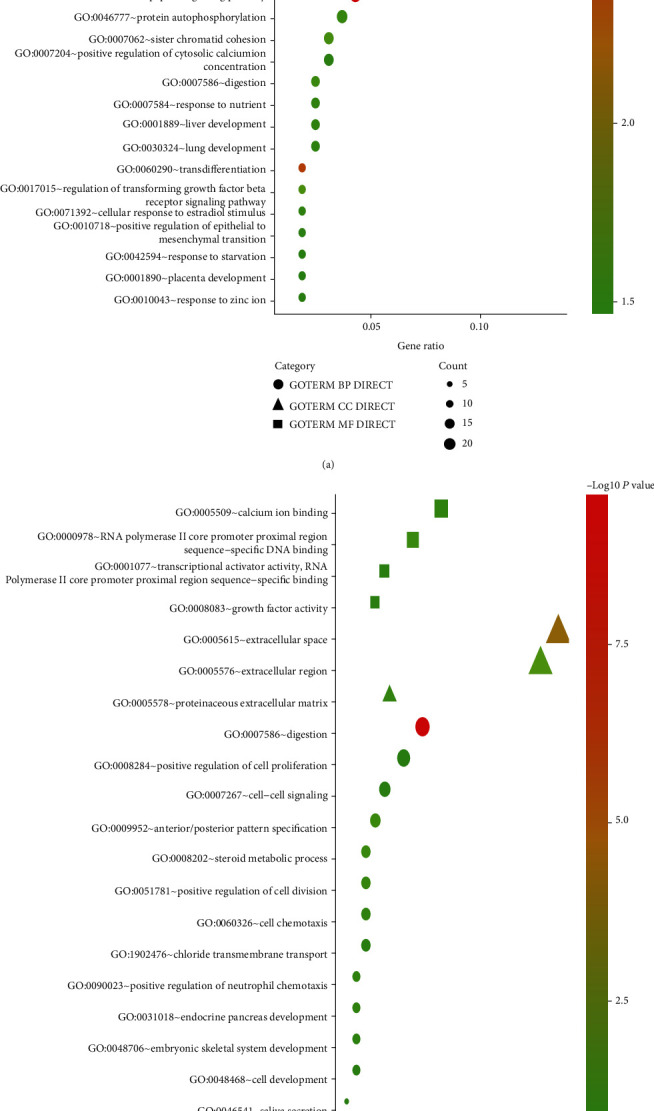
Bubble plot for GO enrichment of DEGs in (a) normal vs. *Hp*-GA and (b) *Hp*-GA vs. *Hp*-GC. The gene ratio is assigned to the *x*-axis and the description of pathway to the *y*-axis. The area of the displayed graphic is proportional to the number of genes assigned to the term, and the color corresponds to the adjusted *P* value.

As shown in Figure 3(a), *Hp*-GA-related DEGs were mainly correlated with the activity of inward rectifying potassium channels, positive regulation of cell proliferation, cell mitosis, digestion, etc. By using KEGG tools, DEGs were mainly enriched in gastric acid secretion and cancer pathways (Table 3(a)).

#### 3.2.2. Functional Enrichment Analysis of *Hp*-GC-Related DEGs

In Figure 3(b), *Hp*-GC-related DEGs were principally associated with digestion, positive regulation of cell proliferation, positive regulation of cell division, and calcium ion binding. By KEGG analysis, DEGs were prevailingly enriched in salivary secretion, neuroactive ligand-receptor interactions, and gastric acid secretion (Table 3(b)).

### 3.3. Construction of the lncRNA-TF-mRNA Regulatory Network

#### 3.3.1. lncRNA-TF-mRNA Regulatory Network of *Hp*-GA-Related DEGs

**Figure 4:**
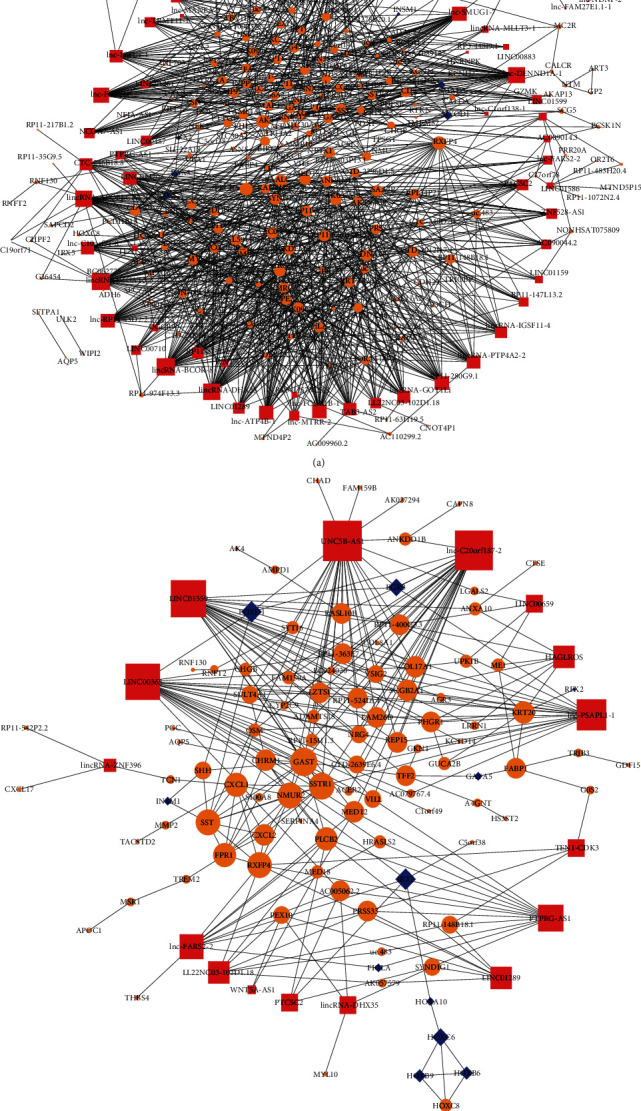
lncRNA-TF-mRNA regulatory networks of (a) *Hp*-GA and (b) Hp-GC. Pink squares indicate lncRNAs, blue diamonds indicate TFs, orange circles indicate target genes, and size increases with the degree.

The lncRNA-TF-mRNA regulatory network of *Hp*-GA contained 63 lncRNAs, 12 TFs, and 209 mRNAs (Figure 4(a)). After sorting by degree, the core of the network was obtained including the top 5 lncRNAs, the top 3 TFs, and the top 10 mRNAs. They were lincRNA-BCOR-8, lincRNA-MGAT5-3, lincRNA-SLC34A2, lincRNA-DHX35, lincRNA-APOBEC3A, CDX2, ETV2, MYOD1, RXFP4, AKT1, PLCB2, BAALC, SAA3P, AC005062.2, DNALI1, MED18, RP11-570H19.2, and SYNDIG1.

#### 3.3.2. lncRNA-TF-mRNA Regulatory Network of *Hp*-GC-Related DEGs

As shown below, the lncRNA-TF-mRNA regulatory network of GC was built including 16 lncRNAs, 11 TFs, and 92 mRNAs (Figure 4(b)). After sorting by degree, the central network was acquired, making up the top 5 lncRNAs, the top 3 TFs, and the top 10 mRNAs. They were UNC5B-AS1, lnc-C20orf187-2, LINC01559, LINC00365, lnc-PSAPL1-1, IRX2, FOXD1, HOXC6, GAST, SSTR, NMUR2, SST, RXFP4, FPR1, CXCL1, PLCB2, KRT20, and CHRM1.

### 3.4. Construction of the ceRNA Regulatory Network

#### 3.4.1. ceRNA Regulatory Network of *Hp*-GA-Related DEGs

**Figure 5:**
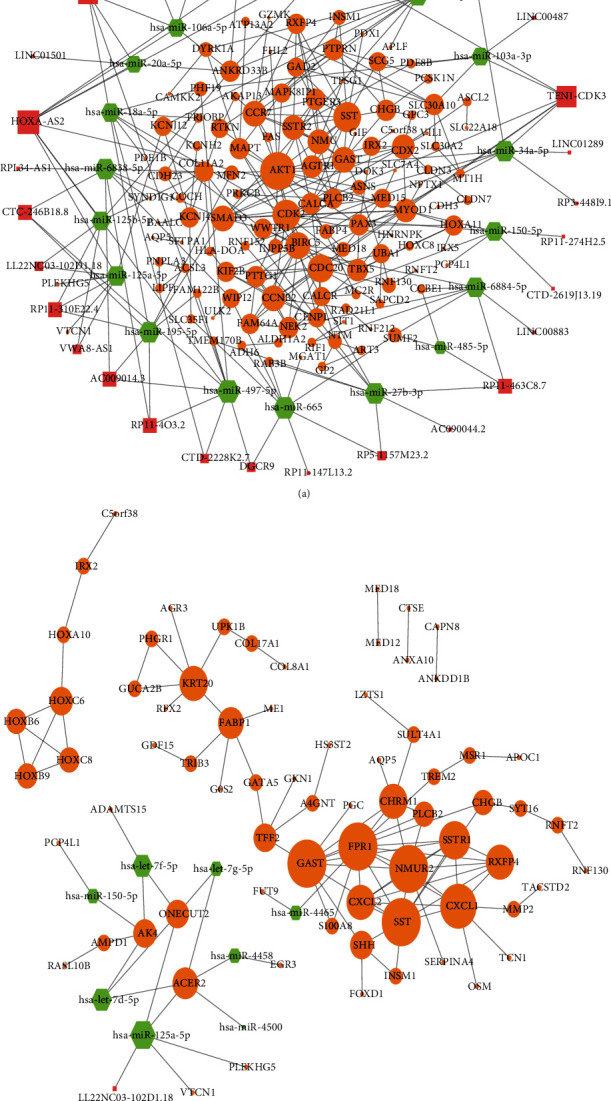
ceRNA regulatory networks of (a) *Hp*-GA and (b) Hp-GC. Pink squares indicate lncRNAs, green hexagons indicate miRNAs, orange circles indicate target genes, and size increases with the degree.

After screening and matching in the miRWalk dataset and DIANA-tools, an integrated lncRNA-miRNA-mRNA network of *Hp*-GA was obtained, including 120 mRNAs, 18 miRNAs, and 27 lncRNAs (Figure 5(a)). By calculating the number of interactions for each RNA, we obtained the top 2 lncRNAs, top 3 miRNAs, and top 10 mRNAs in the network, which were HOXA-AS2, RP11-64C1.1, hsa-miR-497-5p, hsa-miR-665, hsa-miR-145-5p, AKT1, CDK2, SST, CDC20, BIRC5, SMAD3, CCR7, CCNB2, GAST, and CDX2. They were the central lncRNAs, miRNAs, and mRNAs of the network.

#### 3.4.2. ceRNA Regulatory Network of *Hp*-GC-Related DEGs

Using the same method above, the lncRNA-miRNA-mRNA network of *Hp*-GC including 72 mRNAs, 8 miRNAs, and 1 lncRNA was gained (Figure 5(b)). The top 3 gained miRNAs were hsa-miR-125a-5p, hsa-let-7d-5p, and hsa-let-7f-5p. The top 10 gained mRNAs were FPR1, SST, GAST, NMUR2, CXCL1, SSTR1, CXCL2, CHRM1, RXFP4, and KRT20. LL22NC03-102D1.18 was chosen as the only lncRNA. The above genes were the central molecules of the network.

### 3.5. Integration of the Cross-Network

#### 3.5.1. Cross-Network of *Hp*-GA-Related DEGs

**Figure 6:**
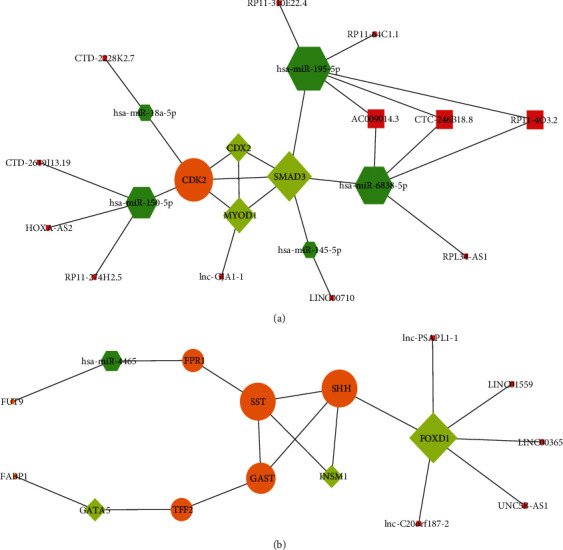
Cross-networks of (a) *Hp*-GA and (b) *Hp*-GC. Pink squares indicate lncRNAs, green hexagons indicate miRNAs, yellow diamonds indicate TFs, orange circles indicate target genes, and size increases with the degree.

Intersecting the above two networks was able to gain a cross-network. 1 DEG, 3 TFs, 12 lncRNAs, and 5 miRNAs were included, which were CDK2, CDX2, SMAD3, MYOD1, RP11-4O3.2, AC009014.3, HOXA-AS2, lnc-GJA1-1, RPL34-AS1, RP11-64C1.1, RP11-310E22.4, CTC-246B18.8, LINC00710, CTD-2228K2.7, CTD-2619J13.19, RP11-274H2.5, hsa-miR-6838-5p, hsa-miR-195-5p, hsa-miR-145-5p, hsa-miR-18a-5p, and hsa-miR-150-5p (Figure 6(a)).

#### 3.5.2. Cross-Network of *Hp*-GC-Related DEGs

To obtain a cross-work of *Hp*-GC-related DEGs, regulatory networks were intersected including 7 DEGs, 3 TFs, 5 lncRNAs, and 1 miRNA. They were FABP1, FPR1, TFF2, GAST, SST, FUT9, SHH, FOXD1, GATA5, INSM1, lnc-C20orf187-2, lnc-PSAPL1-1, UNC5B-AS1, LINC01559, LINC00365, and hsa-miR-4465 (Figure 6(b)). Although no mRNA was found to be coregulated in both lncRNA-TF-mRNA and ceRNA, some regulatory pathways were found to be meaningful.

### 3.6. Validation of DEGs and TFs

**Figure 7:**
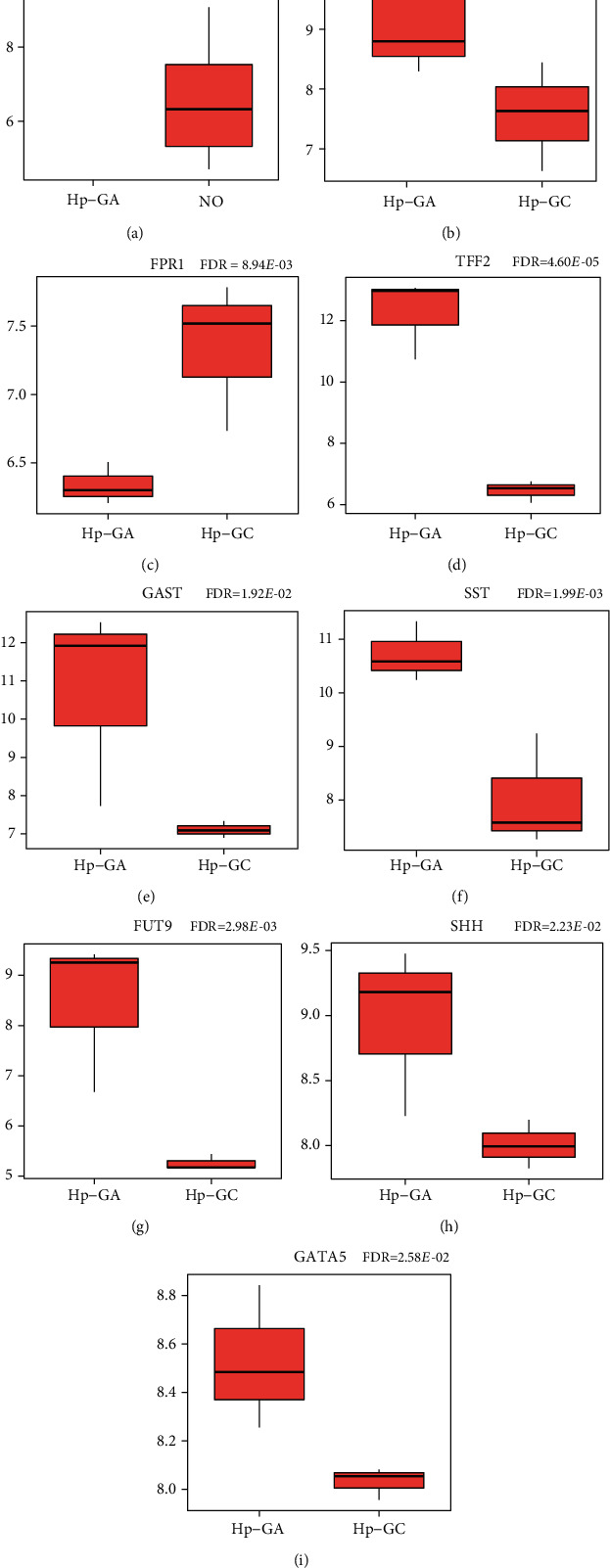
(a) Validation of DEGs and TFs. Validation of DEGs and TFs related to *Hp*-GA in the GSE27411 dataset. (b–i) Validation of DEGs and TFs related to *Hp*-GC in the GSE116312 dataset.

To confirm the analysis results, the GSE27411 dataset was used to verify the above 4 DEGs and TFs related to *Hp*-GA. In consequence, the differentially expressed CDX2 in NO vs. *Hp*-GA was statistically significant while the expression trend was consistent with the screening results (Figure 7(a)). However, CDK2, SMAD3, and MYOD1 were not verified in the GSE27411 dataset. Besides, 10 DEGs and TFs related to *Hp*-GC were confirmed by the GSE116312 dataset. The results revealed that the expression trends of 6 DEGs and GATA5 were the same as in the screening results. FABP1 expressed differences among groups while the trend was the opposite (Figures 7(b)–7(i)). However, FOXD1 and INSM1 were not validated in the GSE116312 dataset.

## 4. Discussions

Exploring DEGs with *Hp*-diseases including GA/GC and their noncoding regulation is of great significance for the early diagnosis and prevention of *Hp*-related gastric diseases. In this research, we constructed lncRNA-TF-mRNA and ceRNA regulatory networks and furthermore comprehensively analyzed the interaction among the network molecules. Our study will help to clear the molecular basis of *Hp*-infected gastric diseases as well as to inform the diagnosis and prevention of *Hp*-infected GC.

Functional analysis of DEGs showed positive regulation of cell proliferation, cell mitosis, and digestion, all related to *Hp*-GA and *Hp*-GC. DEGs of *Hp*-GA were also correlated with inward rectifying potassium channel activity. Besides, calcium ion binding and other functions were linked to *Hp*-GC. Simultaneously, KEGG enrichment analysis showed that *Hp*-GA-related DEGs were mainly associated with gastric acid secretion and cancer pathways, while *Hp*-GC ones were mainly involved in salivary secretion, neuroactive ligand-receptor interactions, and gastric acid secretion. Studies have mentioned that acid secretion had the most significant effect on the development of gastric disorders [29]. Our research displayed that the changes in acid secretion accompanied the process from GA to GC. Thus, the genes involved in this regulation may be closely correlated with the development of gastric diseases.

lncRNAs serve as signals, bait, guide, or scaffold molecules [10]. Among them, by directing TF to the promoter region, lncRNAs play a vital role in gene regulation [30]. Based on this, we constructed lncRNA-TF-mRNA networks. In the *Hp*-GA network, CDX2 has been reported to be a core TF [31], which played a key role in IM and cancer [32]. ETV2 and MYOD1 were key TFs involved in vascular endothelial differentiation, angiogenesis, and myogenic differentiation of bone marrow mesenchymal progenitor cells [33, 34]. However, those five lncRNAs, including lincRNA-BCOR-8, lincRNA-MGAT5-3, lincRNA-SLC34A2, lincRNA-DHX35, and lincRNA-APOBEC3A, have not been reported yet. In the *Hp*-GC network, IRX2, FOXD1, and HOXC6 affected the promotion of proliferation and invasion through transcriptional regulation [35–37]. Some reports showed that UNC5B-AS1 promoted thyroid papillary cancer [38]. LINC01559 hindered YAP phosphorylation and accelerated the pancreatic cancer development [39]. LINC00365 was involved in colorectal cancer by mediating the Wnt/*β*-catenin signaling pathway [40]. There has been no research about lnc-C20orf187-2 and lnc-PSAPL1-1. The relationship between lncRNA and TF found in this study has not been reported. Also, we found that there were two overlapping mRNAs in the *Hp*-GA and *Hp*-GC networks named RXFP4 and PLCB2. Studies have reported that RXFP4 was involved in the regulation of human neuroendocrine tumors [41, 42]. Low expression of PLCB2 can change the RAS/Raf/MAPK signaling pathway, reduce cell viability, promote apoptosis, and inhibit tumorigenesis [43]. However, what role has these two molecules played in the process of NO-GA-GC remains poorly understood.

By constructing the ceRNA network, we screened lncRNAs, miRNAs, and mRNAs involved in the gastric diseases. In the *Hp*-GA network, hsa-miR-497-5p is shown to be lowly expressed in colorectal cancer [15]. It regulated the TGF-*β* signaling pathway, which can lead to cell cycle arrest by regulating SMAD3 [44]. hsa-miR-665, downregulated in gastric signet-ring cell carcinoma and upregulated in gastric adenocarcinoma, may be associated with the invasion and metastasis in signet-ring cell carcinoma [45]. hsa-miR-145-5p was reduced in *Hp*-negative GC patients [46] and could downexpress SOX2, a gastric-type differentiation factor [47]. The relationships between the lncRNAs and the above three miRNAs have not been investigated yet. In addition, HOXA-AS2 was demonstrated in promoting cell proliferation, inducing epithelial-mesenchymal transition in hepatocellular carcinoma via the miR-520c-3p/gPC3 axis [48]. RP11-64C1.1 might be valuable for future investigation. Besides, in the *Hp*-GC-related network, hsa-miR-125a-5p was described to upregulate CCR7 and promote the development of squamous carcinoma in the head or neck [49]. The relationship between hsa-let-7d-5p and tumor has not been specifically reported, while some scholars found it to be closely related to cell senescence [50]. The only lncRNA in the network, LL22NC03-102D1.18, remains to be explored. Also, we found two overlapping mRNAs in two ceRNA networks named SST and GAST. Pieces of research showed the SST affected tumor growth by inhibiting cell proliferation and secretion and inducing apoptosis [51]. It was linked to the invasion and metastasis of the tumor [52]. GAST not only increases the size of gastrointestinal tumors but also inhibits goblet cell differentiation and tumor cell apoptosis [53, 54]. However, how they are regulated by ncRNA was still unknown.

And then, we integrated the lncRNA-TF-mRNA and ceRNA regulatory networks into a cross-network. In the *Hp*-GA-related cross-network, the core mRNAs were CDK2, CDX2, MYOD1, and SMAD3, regulated by different miRNAs and lncRNAs, respectively. CDK2 is a negative regulator of T cells. TGF-*β*-SMAD3 can inhibit CDK2 to promote Treg differentiation [55], which indicates that the immune microenvironment may play an essential role in the gastric diseases. CDX2, MYOD1, and SMAD3 have been demonstrated to participate in the transcription of gastric differentiation. The above results showed that the regulatory network composed of immune and differentiation genes, together with miRNA and lncRNA, played a vital role in the development of *Hp*-GA. An in-depth study of these molecules may reveal the mechanism of *Hp*-GA. In the *Hp*-GC-related cross-network, the core molecules were SST, SHH, and GAST, which contacted with the regulation of neuroendocrine hormones. SHH was described to target INSM1 and promote the progress of lung cancer [56]. The SHH signaling pathway is also activated by the FOXD1, an essential role in the development of the disease [57]. According to this, we could find that immune regulation and differentiation were important in the process of NO-GA, while neuroendocrine regulation was mainly related to the process of GA-GC. Therefore, an in-depth exploration of these molecules will enable us to understand the multistep process of gastric diseases. Using GSE27411 and GSE116312 datasets for further validation, we discovered that in differential genes between NO and *Hp*-GA, CDX2 played an unignorable role. Its function in *Hp*-GA is worthy of further discussion. In the identification of differential genes between *Hp*-GA and *Hp*-GC, FPR1, TFF2, GAST, SST, FUT9, SHH, and GATA5 were assumed significant. Expression differences of FABP1 were shown in the validation datasets while the expression trend above was the opposite. How does FABP1 play in the progression of gastric diseases? It needs to be further studied.

## 5. Conclusion

In summary, in this study, we screened differentially expressed mRNAs and their long noncoding RNA regulatory network with *Hp*-associated diseases including GA and GC. We constructed lncRNA-TF-mRNA, ceRNA, and cross-networks involved in these diseases. Our study might deepen the understanding of *Hp*-related gastric diseases, extend the perception of noncoding regulatory mechanisms, and improve the early diagnosis and prevention of GC.

## Figures and Tables

**Table 1a:** (a) The five most significantly down- and upregulated lncRNAs in normal vs. *Hp*-GA

Gene symbol	Regulation	Log_2_FC	FDR
lnc-DENND1A-1	Down	5.51	2.77*E*-04
lincRNA-SLC34A2	Down	4.50	6.44*E*-04
RP11-310E22.4	Down	4.45	1.19*E*-03
lincRNA-DHX35	Down	4.42	1.33*E*-04
RPL34-AS1	Down	4.34	1.90*E*-03
LINC01586	Up	2.10	1.14*E*-04
RP11-4O3.2	Up	2.19	2.22*E*-04
HOXA-AS2	Up	2.23	4.32*E*-03
lnc-C1QTNF8-1	Up	2.40	5.46*E*-03
AC009014.3	Up	3.54	3.07*E*-04

**Table 1b:** (b) The five most significantly down- and upregulated lncRNAs in *Hp*-GA vs. *Hp*-GC

Gene symbol	Regulation	Log_2_FC	FDR
UNC5B-AS1	Down	3.51	3.38*E*-06
lnc-PSAPL1-1	Down	3.17	2.06*E*-02
SOX21-AS1	Down	2.77	1.37*E*-03
lnc-C20orf187-2	Down	2.53	1.51*E*-05
LINC01133	Down	2.45	8.52*E*-05
HAGLROS	Up	2.35	1.24*E*-02
LINC01289	Up	2.38	1.92*E*-03
LL22NC03102D1.18	Up	2.43	6.03*E*-03
nc-HOXC10-120	Up	2.48	3.22*E*-02
LINC00659	Up	2.94	9.29*E*-04

**Table 2a:** (a) The five most significantly down- and upregulated DEGs in normal vs. *Hp*-GA

Gene symbol	Regulation	Log_2_FC	FDR
PGA3	Down	7.00	3.05*E*-03
NONHSAT006763	Down	6.14	5.24*E*-04
C5orf38	Down	5.94	6.26*E*-07
AC005062.2	Down	5.58	1.45*E*-04
NM_022658	Down	5.33	1.99*E*-04
PDX1	Up	3.65	2.74*E*-05
NMU	Up	3.80	2.66*E*-06
C11orf86	Up	4.37	1.71*E*-08
GP2	Up	5.68	3.00*E*-04
GAST	Up	6.02	1.60*E*-05

**Table 2b:** (b) The five most significantly down- and upregulated DEGs in *Hp*-GA vs. *Hp*-GC

Gene symbol	Regulation	Log_2_FC	FDR
GAST	Down	6.48	8.53*E*-09
SST	Down	5.72	1.08*E*-03
SYT16	Down	5.08	2.22*E*-08
UPK1B	Down	4.34	6.89*E*-05
C11orf86	Down	4.21	4.97*E*-05
MSR1	Up	3.83	1.54*E*-05
C5orf38	Up	3.89	6.91*E*-04
FOXD1	Up	3.93	1.33*E*-04
NM_022658	Up	4.13	2.96*E*-04
S100A8	Up	4.96	2.01*E*-03

**Table 3a:** (a) KEGG enrichment analysis for the DEG-related *Hp*-GA

ID	Description	Ratio	*P* value	Count
hsa04971	Gastric acid secretion	5/105	0.66*E*-02	5
hsa04940	Type I diabetes mellitus	4/105	0.91*E*-02	4
hsa05200	Pathways in cancer	10/105	1.90*E*-02	10
hsa04725	Cholinergic synapse	5/105	2.73*E*-02	5
hsa04110	Cell cycle	5/105	3.87*E*-02	5
hsa05143	African trypanosomiasis	3/105	4.52*E*-02	3

**Table 3b:** (b) KEGG enrichment analysis for the DEG-related *Hp*-GC

ID	Description	Ratio	*P*value	Count
hsa04970	Salivary secretion	4/64	1.43*E*-02	4
hsa04080	Neuroactive ligand-receptor interaction	6/64	2.34*E*-02	6
hsa04971	Gastric acid secretion	3/64	6.99*E*-02	3

## Data Availability

The microarray data used to support the findings of this study are included within the article.

## Supplementary Materials

Supplementary 1 Supplementary file 1 contains 371 differentially expressed genes including 81 upregulated and 290 downregulated genes and compares *Hp*-positive atrophic gastritis with normal gastric mucosal tissues.

Supplementary 2 Supplementary file 2 contains 173 differentially expressed genes including 84 upregulated and 89 downregulated genes and compares *Hp*-positive gastric cancer with *Hp*-positive atrophic gastritis.
