# Supplementary material for: Differentially Expressed mRNAs and Their Long Noncoding RNA Regulatory Network with Helicobacter pylori-Associated Diseases including Atrophic Gastritis and Gastric Cancer
Source: Biomed Res Int. 2020 Nov 17;2020:3012193. doi: 10.1155/2020/3012193 (PMC7686847; doi:10.1155/2020/3012193)
Supplement: Supplementary 1 — Supplementary file 1 contains 371 differentially expressed genes including 81 upregulated and 290 downregulated genes and compares Hp-positive atrophic gastritis with normal gastric mucosal tissues. [file 3012193.f1.docx]

| gene | adj.P.Val | logFC |
| --- | --- | --- |
| LINC01281 | 1.74E-03 | -3.01271 |
| G43172 | 1.49E-03 | -2.33186 |
| TCEAL3 | 2.41E-03 | -3.09794 |
| TSPAN4 | 1.79E-02 | -2.19377 |
| PDE8B | 1.46E-04 | 2.279093 |
| WIPI2 | 5.22E-03 | -2.22569 |
| RGS19 | 9.00E-03 | -2.16401 |
| TXNDC2 | 7.59E-03 | -2.74837 |
| C17orf62 | 4.41E-03 | -2.24011 |
| SLC52A1 | 5.36E-03 | -2.22197 |
| PLEKHG4 | 2.07E-03 | -2.79751 |
| ART3 | 1.56E-05 | 2.465525 |
| IRX2 | 6.89E-06 | -4.96452 |
| SIGIRR | 1.67E-03 | -2.53263 |
| DOK3 | 1.82E-02 | -2.05408 |
| ZNF805 | 3.37E-03 | -2.45361 |
| SUMF2 | 4.59E-02 | -2.02555 |
| GPC3 | 1.92E-03 | -2.1233 |
| SFTPA1 | 1.65E-03 | -2.54791 |
| BIRC5 | 2.59E-02 | 2.045142 |
| CALCA | 3.65E-03 | -3.03658 |
| CALCR | 8.92E-04 | -2.16533 |
| CDH3 | 3.93E-03 | -2.15271 |
| PEX10 | 1.70E-03 | -3.75841 |
| KCNJ4 | 3.11E-03 | -2.9457 |
| MED18 | 4.93E-04 | -3.56693 |
| SLC30A2 | 1.17E-02 | -2.07274 |
| RNFT2 | 5.38E-04 | -4.8749 |
| CDK2 | 9.69E-04 | -3.36055 |
| RNF212 | 2.93E-03 | -2.18358 |
| C19orf48 | 4.53E-04 | -5.24502 |
| RP11-834C11.6 | 1.25E-04 | -2.07849 |
| FAS | 3.73E-03 | -2.5368 |
| CLDN7 | 1.58E-04 | 2.996244 |
| RP4-798A17.5 | 1.90E-02 | 2.028501 |
| ANKRD45 | 3.39E-04 | -3.14259 |
| ULK2 | 3.74E-04 | -2.93239 |
| CENPL | 9.90E-04 | -3.57394 |
| G36454 | 1.12E-02 | -2.09988 |
| WDR45BP1 | 4.60E-03 | -2.10646 |
| CCR7 | 4.25E-02 | -2.21948 |
| KIF2B | 3.80E-03 | -2.47807 |
| RP11-147L13.2 | 8.35E-04 | -2.67118 |
| AK090911 | 8.75E-04 | -2.0582 |
| NPTX1 | 6.65E-03 | -2.19818 |
| FAM64A | 3.30E-03 | 2.194019 |
| RP11-283C24.1 | 1.26E-04 | 2.003859 |
| KCNJ12 | 2.31E-03 | -2.52289 |
| lnc-FOXN1-2 | 4.80E-03 | -2.54752 |
| ADAP2 | 2.79E-03 | -2.10766 |
| C17orf78 | 3.46E-04 | 2.071447 |
| GAST | 1.60E-05 | 6.019561 |
| NAGS | 1.42E-03 | 2.206553 |
| MAPT | 6.85E-03 | -2.84168 |
| SSTR2 | 5.72E-03 | 2.037507 |
| TEN1-CDK3 | 2.77E-04 | -3.98669 |
| MC2R | 3.21E-02 | -2.38071 |
| CCBE1 | 1.06E-03 | -3.35124 |
| RNF152 | 1.17E-05 | -2.55591 |
| BOD1L2 | 1.10E-03 | -2.73455 |
| RP11-35G9.5 | 4.17E-03 | -2.13406 |
| NEK2 | 4.73E-03 | 2.191196 |
| SLC25A41 | 1.11E-02 | 2.372129 |
| SLC30A10 | 7.51E-06 | 2.208686 |
| C1orf140 | 2.48E-03 | -2.18586 |
| lnc-IGFL4-1 | 3.07E-03 | -3.41296 |
| ZNF528-AS1 | 8.69E-04 | -2.39389 |
| C19orf71 | 1.54E-02 | -2.24776 |
| SNAPC2 | 2.93E-03 | -2.093 |
| OR7E25P | 2.81E-04 | 2.085414 |
| CYP4F8 | 2.77E-03 | -2.00299 |
| ETV2 | 3.31E-03 | -4.40808 |
| NONHSAT066698 | 4.18E-03 | -2.91434 |
| AC008753.4 | 5.51E-03 | -2.60571 |
| CTD-2619J13.19 | 2.89E-03 | -3.11623 |
| LINC00487 | 3.07E-03 | -3.21361 |
| lnc-THADA-4 | 3.55E-03 | -2.88432 |
| SH3BP5L | 1.70E-03 | -3.22633 |
| RTKN | 4.28E-03 | -2.42935 |
| LINC01159 | 3.66E-07 | -3.01835 |
| AC104655.2 | 4.50E-03 | -2.12295 |
| FHL2 | 3.57E-04 | -2.96221 |
| NONHSAT000338 | 6.63E-03 | -2.15295 |
| AF116618 | 1.53E-03 | -2.57768 |
| PTPRN | 2.04E-02 | 2.32398 |
| PAX3 | 6.83E-03 | -3.87814 |
| ABCG8 | 1.40E-02 | 2.269758 |
| MFN2 | 5.65E-04 | -2.18703 |
| MTND4P25 | 2.78E-04 | 2.2164 |
| MIIP | 4.37E-03 | -2.04748 |
| AC018892.7 | 3.78E-03 | -2.16779 |
| AC007248.7 | 3.81E-03 | -2.12292 |
| C1orf158 | 2.20E-03 | -2.34591 |
| AC062016.2 | 2.27E-04 | 2.182359 |
| RIF1 | 5.04E-03 | -2.4763 |
| TMEM82 | 1.30E-02 | -2.49529 |
| NONHSAT075809 | 4.32E-04 | 2.108592 |
| AC009960.2 | 1.17E-02 | -2.21271 |
| VIL1 | 2.19E-03 | 2.518953 |
| ATP13A2 | 3.47E-03 | -2.6491 |
| ACSL3 | 8.48E-04 | -2.52932 |
| RP11-315F22.1 | 6.71E-04 | -2.49514 |
| AC110299.2 | 8.27E-03 | -2.00991 |
| RP11-223J15.2 | 1.97E-03 | -2.35677 |
| RAD21L1 | 2.28E-04 | -2.5413 |
| CHGB | 1.14E-02 | 2.155886 |
| INSM1 | 2.12E-02 | 2.455486 |
| SYNDIG1 | 3.53E-04 | -4.18382 |
| AP001065.7 | 2.86E-02 | -2.16778 |
| KRTAP20-2 | 2.73E-03 | -2.15117 |
| DYRK1A | 7.68E-05 | 2.088941 |
| AK026868 | 7.54E-04 | -2.81435 |
| BC062773 | 1.82E-06 | 2.037961 |
| SLC7A4 | 3.07E-06 | 2.660773 |
| LL22NC03-102D1.18 | 7.60E-04 | -3.27888 |
| DGCR9 | 8.05E-03 | -2.28884 |
| MED15 | 1.07E-03 | -2.7441 |
| CRIP1P4 | 3.77E-04 | 2.081334 |
| DNALI1 | 6.14E-04 | -2.59407 |
| TRIOBP | 3.98E-04 | -4.68376 |
| PNPLA3 | 5.03E-03 | -2.0822 |
| TBC1D5 | 4.12E-03 | -4.53963 |
| NONHSAT089187 | 1.84E-03 | -2.0074 |
| PTPRG-AS1 | 1.76E-03 | -3.07379 |
| CDC20 | 1.44E-03 | 2.254228 |
| RP11-274H2.5 | 1.59E-03 | -2.0818 |
| WWTR1 | 2.29E-04 | -2.60303 |
| RP11-656A15.1 | 2.16E-03 | -2.84147 |
| RP11-12K11.2 | 1.10E-03 | -2.2912 |
| RP11-373E16.4 | 6.05E-03 | -2.10258 |
| MTND5P15 | 6.84E-05 | 2.044203 |
| SST | 8.99E-04 | 3.176659 |
| lnc-APOD-6 | 1.60E-03 | -3.19223 |
| AC090044.2 | 9.98E-05 | 2.026259 |
| OGG1 | 1.66E-03 | -3.3444 |
| IL17RE | 1.83E-03 | -2.36132 |
| CRIP1P2 | 4.77E-04 | 2.011962 |
| RP5-1157M23.2 | 1.52E-03 | -2.47622 |
| RP11-64C1.1 | 5.82E-03 | -3.48377 |
| WNT5A-AS1 | 1.10E-03 | -3.53045 |
| CTD-2185K10.1 | 4.65E-03 | -2.33478 |
| LINC00883 | 3.66E-03 | -2.50981 |
| UPK1B | 2.21E-03 | 3.078975 |
| COL6A4P2 | 3.45E-03 | -3.56222 |
| RP11-91K8.4 | 2.47E-03 | -2.12389 |
| AGTR1 | 9.77E-04 | -2.62999 |
| AK090977 | 8.98E-04 | -2.83934 |
| MFSD10 | 1.89E-03 | -3.21485 |
| FGFBP1 | 3.28E-03 | 3.426933 |
| RP11-17E2.2 | 4.76E-03 | -2.30214 |
| NMU | 2.66E-06 | 3.795363 |
| RP11-63H19.5 | 1.74E-03 | -2.05153 |
| ADH6 | 2.67E-02 | 2.640663 |
| NONHSAT003969 | 1.01E-02 | -2.77484 |
| RPL34-AS1 | 1.90E-03 | -4.33744 |
| lnc-NDNF-2 | 9.19E-03 | 2.077239 |
| RP11-4O3.2 | 2.22E-04 | 2.189967 |
| CTD-2012I17.1 | 3.74E-04 | -3.3155 |
| RP11-148B18.1 | 9.00E-04 | -3.31235 |
| HMGB3P10 | 3.65E-03 | -2.43941 |
| SDHAP3 | 1.90E-03 | -2.04409 |
| CTC-340D7.1 | 7.66E-04 | -2.91072 |
| RP11-1072N2.4 | 9.54E-04 | 2.016978 |
| CTD-2278B20.1 | 3.12E-03 | 3.090307 |
| lnc-ST8SIA4-2 | 1.15E-03 | -2.65686 |
| AK057579 | 3.90E-04 | -3.55939 |
| AC009014.3 | 3.07E-04 | 3.538665 |
| LINC01470 | 1.07E-03 | -2.27745 |
| RNF130 | 1.53E-04 | -4.75982 |
| MGAT1 | 4.02E-04 | -2.25656 |
| CTD-2228K2.7 | 1.06E-02 | -2.70673 |
| C5orf38 | 6.26E-07 | -5.94378 |
| CTD-2296D1.5 | 3.59E-03 | -2.0905 |
| lnc-MTRR-2 | 2.56E-03 | -2.35069 |
| ANKRD33B | 7.24E-03 | -2.30222 |
| GZMK | 5.70E-03 | -2.1499 |
| FAM159B | 5.72E-03 | 2.635981 |
| RP11-974F13.3 | 1.87E-03 | -2.88992 |
| RP11-346J10.1 | 1.34E-02 | -2.19605 |
| lnc-RP11-43D2.2.1-14 | 2.25E-03 | -2.2236 |
| PCDHB13 | 2.56E-03 | -2.10564 |
| SPINK5 | 9.31E-04 | -2.6602 |
| SPINK13 | 2.44E-04 | -2.34672 |
| PTTG1 | 5.70E-03 | 2.079509 |
| CTC-251I16.1 | 1.96E-02 | -2.30381 |
| HLA-DOA | 2.32E-03 | -2.68141 |
| COL11A2 | 2.08E-03 | -2.63747 |
| DLK2 | 3.65E-03 | -2.54924 |
| RP11-301G19.1 | 3.99E-04 | -2.32713 |
| RP11-14N7.1 | 3.64E-02 | -2.3699 |
| lnc-FCGR1B-1 | 2.78E-03 | -2.04634 |
| NCOA7-AS1 | 2.98E-03 | -2.56718 |
| G43180 | 1.42E-03 | -4.06491 |
| DACT2 | 3.04E-03 | 2.771593 |
| lnc-FARS2-2 | 5.66E-04 | -3.26092 |
| TMEM170B | 1.70E-02 | -2.01529 |
| RP3-448I9.1 | 1.67E-03 | -2.92008 |
| lnc-MANEA-3 | 2.54E-03 | -2.63996 |
| SLC35F1 | 3.72E-02 | -2.11274 |
| lnc-GJA1-1 | 2.99E-05 | -2.1284 |
| NONHSAT006763 | 5.24E-04 | -6.14106 |
| RXFP4 | 2.12E-04 | -3.26876 |
| AC005062.2 | 1.45E-04 | -5.58067 |
| AC004920.3 | 4.70E-03 | -3.51613 |
| MTND4P2 | 2.92E-02 | -2.29622 |
| CLDN3 | 5.39E-03 | 3.431097 |
| ASNS | 1.99E-02 | -2.03496 |
| MYL10 | 3.26E-04 | -4.01595 |
| PCP4L1 | 1.31E-02 | -2.01169 |
| KCNH2 | 6.31E-03 | 2.241293 |
| AC018634.9 | 1.32E-03 | 2.909134 |
| AC003984.1 | 3.07E-03 | -2.37905 |
| OR2A13P | 1.07E-03 | -2.06642 |
| RP11-445N20.2 | 4.43E-04 | -2.02827 |
| CHPF2 | 9.64E-03 | -2.93155 |
| RP11-208G20.2 | 3.06E-02 | 2.754596 |
| NONHSAT124818 | 3.17E-03 | -2.82003 |
| THAP1 | 5.31E-03 | -2.18603 |
| RP11-280G9.1 | 2.09E-03 | -2.20644 |
| FABP4 | 1.37E-02 | -2.6611 |
| OTUD6B-AS1 | 6.46E-03 | -2.02979 |
| RP11-403P13.1 | 4.94E-04 | -2.16398 |
| ZNF703 | 1.16E-05 | 2.643225 |
| LINC01289 | 4.88E-04 | -3.46219 |
| RP11-68L18.1 | 5.24E-03 | -2.17716 |
| BAALC | 1.05E-03 | -2.27131 |
| RP11-570H19.2 | 2.10E-04 | -2.38958 |
| SIT1 | 1.17E-03 | -2.01345 |
| RP11-327I22.5 | 1.68E-03 | -3.03267 |
| lnc-FAM27E1.1-1 | 2.59E-04 | -2.07445 |
| AC129778.2 | 1.79E-04 | 2.075164 |
| HSPBL2 | 4.63E-06 | 2.093585 |
| TRPM6 | 2.23E-03 | -2.38324 |
| HNRNPK | 3.59E-03 | -2.51884 |
| LINC01501 | 5.87E-03 | -2.90506 |
| PTCSC2 | 7.44E-04 | -3.81048 |
| PTPRVP | 1.67E-03 | -3.08896 |
| PHF19 | 4.02E-03 | -2.3235 |
| EDF1 | 1.52E-03 | -2.32285 |
| SAPCD2 | 8.36E-05 | 2.715161 |
| RUSC2 | 4.21E-03 | -2.37898 |
| RP11-374M1.4 | 2.83E-04 | -2.0487 |
| RP11-483H20.4 | 1.37E-04 | 2.512314 |
| PCSK1N | 2.72E-03 | 2.865088 |
| HSPB1P2 | 4.63E-05 | 2.204681 |
| XAGE1A | 2.49E-03 | -2.10407 |
| AY927528 | 8.30E-03 | -2.24537 |
| FAM122B | 5.41E-04 | -5.00615 |
| TAB3-AS2 | 7.04E-04 | -2.3708 |
| UBA1 | 1.42E-03 | -2.0074 |
| PABPC1L2B | 1.53E-03 | -2.5613 |
| RP11-156J23.1 | 2.14E-02 | 2.096087 |
| ARMCX3 | 8.86E-03 | -2.26728 |
| ZNF736P10Y | 1.30E-03 | 2.374558 |
| RP11-66A2.1 | 1.42E-03 | -2.45136 |
| OR2T6 | 3.02E-04 | 2.025858 |
| lnc-C10orf31-8 | 4.30E-04 | -2.4889 |
| LINC00710 | 4.77E-03 | -2.00718 |
| RP11-310E22.4 | 1.19E-03 | -4.44903 |
| GAD2 | 4.97E-03 | -3.03089 |
| INPP5B | 1.08E-02 | -2.26646 |
| AK056830 | 1.70E-03 | -2.46217 |
| CDH23 | 1.18E-03 | -3.17272 |
| LIPF | 2.15E-02 | -4.07392 |
| ASCL2 | 1.69E-03 | 2.15522 |
| SAA3P | 4.83E-04 | -2.90925 |
| GIF | 2.11E-02 | -4.02108 |
| SLC22A24 | 1.12E-02 | -2.0449 |
| RAB3B | 1.37E-06 | 3.492908 |
| MMP10 | 1.23E-04 | 2.4963 |
| AK127883 | 7.48E-03 | -2.74528 |
| PVRL1 | 1.32E-03 | -2.49444 |
| RPL34P21 | 2.22E-05 | 2.236446 |
| NTM | 4.60E-04 | -2.6587 |
| THYN1 | 7.94E-03 | -2.68051 |
| SLC22A18 | 2.56E-03 | -2.45677 |
| MYOD1 | 1.06E-03 | -2.01677 |
| MRGPRX4 | 5.09E-04 | -3.30672 |
| MAPK8IP1 | 2.97E-03 | -2.90236 |
| PGA3 | 3.05E-03 | -6.96279 |
| NFIA-AS1 | 1.93E-02 | -2.89662 |
| C11orf86 | 1.71E-08 | 4.371571 |
| FOLR1 | 3.97E-04 | -2.17202 |
| BC029614 | 1.72E-03 | -2.23378 |
| PTGER3 | 3.27E-02 | -3.20081 |
| RN7SKP15 | 9.97E-03 | -2.54105 |
| TBX5 | 6.17E-04 | 2.105643 |
| CAMKK2 | 9.14E-03 | -2.35996 |
| AQP5 | 2.64E-04 | 3.052337 |
| PDE1B | 2.60E-02 | -2.34879 |
| CNOT4P1 | 9.90E-03 | -2.00802 |
| CDX2 | 1.59E-02 | 2.985109 |
| KDELC1 | 4.33E-03 | -2.44827 |
| lnc-ATP4B-1 | 1.29E-03 | -2.10466 |
| PDX1 | 2.74E-05 | 3.645685 |
| VWA8-AS1 | 2.33E-03 | -3.07156 |
| PRR20A | 8.33E-06 | 2.026299 |
| VTCN1 | 4.69E-06 | 2.176858 |
| LINC00370 | 8.51E-04 | -4.25643 |
| LINC01599 | 4.53E-02 | -2.12689 |
| ZDHHC22 | 9.38E-04 | -3.27906 |
| AKT1 | 4.20E-04 | -4.38563 |
| PLEKHG5 | 1.78E-04 | -3.96583 |
| COCH | 1.91E-03 | -2.36493 |
| NONHSAT037706 | 1.22E-02 | -2.30197 |
| COQ6 | 9.29E-04 | -3.53006 |
| RP11-463C8.7 | 2.38E-03 | -2.23469 |
| lnc-C1orf138-1 | 2.67E-03 | -2.09306 |
| CRIP1 | 5.69E-04 | 2.098381 |
| RP11-12O16.1 | 1.41E-02 | -2.04741 |
| PLCB2 | 6.06E-04 | -4.19905 |
| EXD1 | 3.84E-03 | -3.00308 |
| ALDH1A2 | 3.76E-03 | -3.6019 |
| LCE3E | 1.25E-03 | -3.56112 |
| MTFMT | 1.69E-03 | -2.83648 |
| AL591704.9 | 5.82E-04 | -3.31101 |
| S100A13 | 3.48E-03 | -2.33497 |
| LINC01586 | 1.14E-04 | 2.095487 |
| lnc-SLC35F5-16 | 1.16E-03 | -2.38791 |
| UBE2CP4 | 3.36E-03 | 2.338349 |
| SCG5 | 2.77E-03 | 2.955568 |
| C15orf41 | 5.64E-03 | -2.10416 |
| MGA | 2.12E-03 | -2.75736 |
| DUOXA2 | 1.83E-04 | -3.23466 |
| CCNB2 | 2.02E-02 | 2.287689 |
| FAM189B | 3.49E-03 | -2.54903 |
| SMAD3 | 4.29E-03 | -2.97002 |
| AKAP13 | 6.56E-03 | -2.01567 |
| RP11-217B1.2 | 2.67E-02 | -2.05453 |
| RP11-161M6.2 | 1.90E-04 | 2.496964 |
| lnc-C1QTNF8-1 | 5.46E-03 | 2.398576 |
| TPSG1 | 4.94E-04 | 2.862606 |
| PRSS33 | 8.40E-04 | -3.86628 |
| GP2 | 3.00E-04 | 5.678472 |
| RP11-441F2.5 | 4.02E-03 | -3.43664 |
| BX648197 | 8.54E-04 | -2.77918 |
| TAF1C | 1.54E-04 | -4.38842 |
| SLAMF9 | 1.81E-03 | -3.05448 |
| PRKCB | 5.64E-04 | -3.11864 |
| C16orf82 | 6.84E-04 | -2.00378 |
| IRX5 | 9.33E-05 | -2.1136 |
| CES1P2 | 9.34E-04 | 2.819064 |
| MT1H | 1.76E-02 | -2.31423 |
| CROCCP3 | 1.32E-03 | -3.45917 |
| GPR127 | 6.07E-05 | 2.230437 |
| CTC-246B18.8 | 3.26E-03 | -2.10957 |
| GUSBP11 | 7.01E-04 | -3.0276 |
| nc-HOXC10-120 | 4.62E-04 | -4.41753 |
| HOXA-AS2 | 4.32E-03 | 2.227474 |
| NM_005523 | 9.50E-03 | -2.04535 |
| lincRNA-MGAT5-3 | 3.37E-03 | -2.07911 |
| APLF | 1.93E-03 | -3.5529 |
| lincRNA-DHX35 | 1.33E-04 | -4.41945 |
| lincRNA-IGSF11-4 | 2.11E-04 | -3.8186 |
| lincRNA-SLC34A2 | 6.44E-04 | -4.50266 |
| lnc-TRMT11-1 | 8.81E-03 | -2.04629 |
| lincRNA-HAS2-3 | 7.40E-03 | -2.76604 |
| lnc-DENND1A-1 | 2.77E-04 | -5.5084 |
| lincRNA-BCOR-8 | 4.28E-04 | -2.56535 |
| lincRNA-MPDZ-2 | 1.05E-02 | -2.11221 |
| lincRNA-MLLT3-1 | 1.02E-02 | -2.36459 |
| lincRNA-PTP4A2-2 | 9.54E-04 | -2.47573 |
| lincRNA-GOT1L1 | 1.15E-03 | -2.21923 |
| lincRNA-APOBEC3A | 5.87E-04 | -2.99746 |
| NM_022658 | 1.99E-04 | -5.33095 |
| lnc-SMUG1-7 | 3.39E-03 | -2.89105 |
| uc.467 | 7.41E-03 | -2.33481 |
| uc.473 | 9.65E-03 | -2.04533 |
| uc.10 | 6.73E-03 | -2.01976 |
| uc.57 | 2.42E-03 | -2.64195 |
| uc.483 | 8.46E-04 | -3.32422 |
