# Supplementary material for: Differentially Expressed mRNAs and Their Long Noncoding RNA Regulatory Network with Helicobacter pylori-Associated Diseases including Atrophic Gastritis and Gastric Cancer
Source: Biomed Res Int. 2020 Nov 17;2020:3012193. doi: 10.1155/2020/3012193 (PMC7686847; doi:10.1155/2020/3012193)
Supplement: Supplementary 2 — Supplementary file 2 contains 173 differentially expressed genes including 84 upregulated and 89 downregulated genes and compares Hp-positive gastric cancer with Hp-positive atrophic gastritis. [file 3012193.f2.docx]

| gene | adj.P.Val | logFC |
| --- | --- | --- |
| IRX2 | 3.58E-03 | 2.854004 |
| PEX10 | 3.73E-02 | 2.255332 |
| MED18 | 1.93E-02 | 2.192589 |
| EFS | 4.75E-06 | -2.34568 |
| RNFT2 | 1.49E-02 | 3.102158 |
| CTSE | 4.94E-03 | -2.27726 |
| C19orf48 | 3.03E-02 | 3.137707 |
| FAM3B | 1.49E-04 | -2.40989 |
| KRT20 | 4.34E-04 | -3.24303 |
| CHAD | 1.58E-02 | -2.1622 |
| BC024020 | 1.48E-04 | 2.010744 |
| CD300C | 8.00E-04 | 2.074814 |
| RASL10B | 7.34E-04 | 2.175742 |
| GAST | 8.53E-09 | -6.47629 |
| TEN1-CDK3 | 1.96E-02 | 2.234831 |
| CCBE1 | 2.26E-02 | 2.432438 |
| ONECUT2 | 9.62E-03 | 3.26219 |
| RP11-15I11.3 | 2.28E-04 | -2.30479 |
| RFX2 | 8.83E-06 | 2.094933 |
| ANGPTL6 | 5.52E-04 | -2.10429 |
| FCGBP | 4.08E-03 | -2.07199 |
| CXCL17 | 6.53E-03 | -2.85447 |
| CAPN8 | 3.72E-03 | -2.26871 |
| FPR1 | 8.19E-03 | 2.450588 |
| GDF15 | 2.23E-03 | 2.179479 |
| ETV2 | 4.45E-02 | 2.494519 |
| APOC1 | 1.10E-03 | 2.440465 |
| GRIN2D | 3.85E-03 | 2.291723 |
| CTD-2639E6.4 | 1.88E-04 | -2.02308 |
| FIGLA | 4.43E-05 | -2.17619 |
| REG1B | 1.21E-02 | 2.179011 |
| FABP1 | 4.20E-02 | 3.134578 |
| GKN1 | 5.31E-03 | -3.27739 |
| GALNT5 | 1.56E-03 | -2.23784 |
| HAGLROS | 1.24E-02 | 2.34861 |
| AC009960.2 | 2.06E-03 | 2.078417 |
| AC079767.4 | 6.40E-03 | 2.361268 |
| ANO7 | 1.68E-04 | -3.11247 |
| CST1 | 3.30E-04 | 2.297453 |
| CELA3B | 7.63E-03 | 3.299785 |
| GATA5 | 1.48E-02 | -2.95777 |
| LINC00659 | 9.29E-04 | 2.937699 |
| TRIB3 | 2.67E-03 | 2.233142 |
| CHGB | 6.69E-04 | -2.22694 |
| lnc-C20orf187-2 | 1.51E-05 | -2.5301 |
| INSM1 | 2.11E-03 | -3.23035 |
| SYNDIG1 | 2.15E-02 | 2.326746 |
| BCAS4 | 3.88E-03 | 2.03098 |
| TFF2 | 7.50E-05 | -3.35734 |
| AP001065.7 | 2.57E-03 | 2.369069 |
| LL22NC03-102D1.18 | 6.03E-03 | 2.42751 |
| OSM | 4.14E-02 | 2.253951 |
| LGALS2 | 5.49E-04 | 2.939388 |
| SULT4A1 | 4.26E-03 | -2.46065 |
| CRIP1P4 | 9.64E-06 | -2.35937 |
| TRIOBP | 3.06E-02 | 2.654786 |
| GUCA2B | 2.84E-02 | -2.09167 |
| PTPRG-AS1 | 2.72E-03 | 2.289719 |
| A4GNT | 5.36E-03 | -2.01951 |
| RARRES1 | 4.41E-03 | 2.02777 |
| SST | 1.08E-03 | -5.71979 |
| LRRN1 | 2.77E-03 | 2.507476 |
| CRIP1P2 | 3.80E-06 | -2.34546 |
| VILL | 7.13E-06 | -2.78447 |
| WNT5A-AS1 | 2.30E-02 | 2.117018 |
| COL8A1 | 1.36E-04 | 3.585125 |
| UPK1B | 6.89E-05 | -4.33739 |
| COL6A4P2 | 4.88E-02 | 2.061243 |
| AK4 | 1.24E-02 | -2.27643 |
| lnc-PSAPL1-1 | 2.06E-02 | -3.17103 |
| FGFBP1 | 3.38E-02 | -2.93156 |
| CXCL2 | 2.90E-02 | 2.902882 |
| CXCL1 | 3.88E-02 | 2.548164 |
| RP11-542P2.2 | 1.64E-02 | -2.03688 |
| RP11-148B18.1 | 8.67E-03 | 2.164763 |
| ANXA10 | 3.69E-04 | -2.34028 |
| FOXD1 | 1.33E-04 | 3.934861 |
| RP11-524L6.4 | 2.65E-04 | -2.56261 |
| AK057579 | 7.11E-03 | 2.120955 |
| NMUR2 | 5.01E-05 | -3.74765 |
| RNF130 | 2.02E-02 | 2.616131 |
| TRIM7 | 3.13E-03 | -2.78791 |
| C5orf38 | 6.91E-04 | 3.891353 |
| FAM159B | 2.08E-03 | -2.4647 |
| ANKDD1B | 2.89E-04 | -2.56977 |
| THBS4 | 1.36E-02 | 3.257676 |
| TREM2 | 6.22E-05 | 2.716085 |
| PGC | 1.48E-02 | -2.04566 |
| ME1 | 3.62E-05 | -2.10234 |
| G36899 | 1.40E-03 | -2.20285 |
| FCGR1C | 9.74E-05 | 2.190106 |
| lnc-FARS2-2 | 1.33E-02 | 2.002306 |
| DPCR1 | 4.85E-03 | -3.33323 |
| FUT9 | 4.07E-03 | -2.92506 |
| FAM26D | 1.84E-03 | -2.33987 |
| RXFP4 | 8.63E-03 | 2.053255 |
| AGR3 | 5.09E-03 | -2.19339 |
| AC005062.2 | 2.39E-02 | 2.989488 |
| MTND4P2 | 9.00E-03 | 2.027204 |
| LINC01133 | 8.52E-05 | -2.45411 |
| MYL10 | 4.75E-02 | 2.071965 |
| SLC26A5 | 2.43E-04 | -2.79231 |
| PCP4L1 | 4.33E-03 | 2.116784 |
| SHH | 9.82E-05 | -2.2652 |
| GCNT1P5 | 4.18E-03 | -2.00608 |
| DEFT1P | 9.85E-04 | 2.345763 |
| MSR1 | 1.54E-05 | 3.832701 |
| LZTS1 | 2.93E-04 | 2.646036 |
| EGR3 | 1.62E-04 | 2.330635 |
| C1orf49 | 1.38E-04 | -2.0451 |
| FAM150A | 8.14E-03 | 2.36131 |
| LINC01289 | 1.92E-03 | 2.381989 |
| BHLHE22 | 6.41E-03 | 2.18607 |
| AK027294 | 3.98E-04 | 2.441051 |
| PTCSC2 | 1.80E-02 | 2.220247 |
| CLIC3 | 4.43E-03 | -2.06214 |
| ACER2 | 1.53E-05 | -2.59135 |
| RP11-363E7.4 | 1.79E-04 | -2.63226 |
| DMRTA1 | 6.73E-03 | -2.85777 |
| G0S2 | 1.91E-03 | 2.081011 |
| MED12 | 8.77E-04 | -3.3237 |
| AKR1C6P | 2.84E-03 | -2.03001 |
| AKR1C7P | 1.00E-02 | -2.08615 |
| UNC5B-AS1 | 3.38E-06 | -3.51189 |
| COL17A1 | 1.81E-04 | -3.30257 |
| AKR1C2 | 9.26E-03 | -2.29342 |
| RP11-400G3.3 | 2.62E-04 | -2.31502 |
| CYP2C9 | 7.88E-05 | -2.30751 |
| TCN1 | 1.65E-02 | -2.57323 |
| CHRM1 | 7.33E-06 | -3.39128 |
| HRASLS2 | 3.72E-06 | -3.60189 |
| KCTD14 | 1.91E-04 | -2.42788 |
| RP4-800M22.4 | 1.80E-02 | 2.080502 |
| GUCY1A2 | 1.05E-02 | 2.109729 |
| VSIG2 | 2.24E-03 | -2.46744 |
| TACSTD2 | 1.68E-02 | -2.25433 |
| SCGB2A1 | 2.94E-05 | -4.17308 |
| C11orf86 | 4.97E-05 | -4.2072 |
| ADAMTS15 | 3.08E-04 | -2.10696 |
| LINC01559 | 2.44E-06 | -2.40629 |
| MGP | 1.38E-04 | 2.282067 |
| REP15 | 1.44E-04 | -3.32434 |
| AQP5 | 1.45E-04 | -3.12929 |
| LINC00365 | 5.69E-03 | -2.37697 |
| AMPD1 | 1.17E-03 | -3.23215 |
| SOX21-AS1 | 1.37E-03 | -2.76709 |
| VTCN1 | 9.19E-06 | -3.18084 |
| PLEKHG5 | 2.05E-02 | 2.228727 |
| SSTR1 | 1.13E-03 | -2.38413 |
| SYT16 | 2.22E-08 | -5.08269 |
| SERPINA4 | 2.98E-02 | -2.14698 |
| CRIP1 | 1.68E-05 | -2.37231 |
| PLCB2 | 1.66E-02 | 2.602053 |
| S100A8 | 2.01E-03 | 4.963355 |
| NRG4 | 5.69E-05 | -2.25504 |
| PHGR1 | 3.03E-03 | -2.57709 |
| CRABP1 | 3.62E-03 | 3.588958 |
| PRSS33 | 3.85E-04 | 3.290264 |
| ZNF319 | 3.21E-03 | -2.72013 |
| TAF1C | 4.39E-03 | 2.721235 |
| HS3ST2 | 1.48E-03 | 2.866929 |
| MMP2 | 6.87E-05 | -2.27554 |
| nc-HOXC10-120 | 3.22E-02 | 2.484259 |
| lincRNA-BLID-2 | 5.03E-03 | -2.18645 |
| lincRNA-ZNF396 | 4.75E-02 | -2.10276 |
| lincRNA-DHX35 | 3.84E-02 | 2.283533 |
| NM_022658 | 2.96E-04 | 4.126336 |
| HOXC6 | 8.99E-05 | 3.69846 |
| HOXB6 | 1.73E-03 | 2.169005 |
| HOXB9 | 1.12E-03 | 2.059391 |
| TIAL1 | 1.15E-04 | 2.205051 |
| HOXA10 | 1.52E-02 | 2.636391 |
| uc.483 | 1.53E-02 | 2.059457 |
